# Prevalence and patterns of electronic cigarette and heated tobacco product use among Italian adults in 2024: A cross-sectional study

**DOI:** 10.18332/tpc/213721

**Published:** 2025-12-15

**Authors:** Marco Scala, Irene Possenti, Alessandra Lugo, Anna Odone, Luc Smits, Silvano Gallus

**Affiliations:** 1 Department of Medical Epidemiology, Istituto di Ricerche Farmacologiche Mario Negri IRCCS, Milan, Italy; 2 Department of Public Health Experimental and Forensic Medicine, School of Public Health, University of Pavia, Pavia, Italy; 3 Medical Direction, Fondazione IRCCS Policlinico San Matteo, Pavia, Italy; 4 Department of Epidemiology, Care and Public Health Research Institute, Maastricht University, Maastricht, Netherlands

**Keywords:** ENDS, electronic cigarettes, heated tobacco products, smoking, dual use

## Abstract

**INTRODUCTION:**

Electronic cigarettes (ECs) and heated tobacco products (HTPs) have gained popularity worldwide, despite concerns about their safety. In Italy, updated national data on these devices remain scarce. The aim of this study is to estimate prevalence, patterns, determinants and trends of EC and HTP use among Italian adults in 2024.

**METHODS:**

A face-to-face cross-sectional survey was conducted in 2024 on a representative sample of 3125 Italian individuals aged ≥15 years. Data were collected through interviewer-administered questionnaires and included self-reported information on conventional cigarette (CC), EC, and HTP use. We used multivariable logistic regression models to assess adjusted odds ratios (AOR) for EC and HTP use, including sex, age, education level, and smoking status as covariates. Trends (2022–2024) were assessed using analogous surveys.

**RESULTS:**

In 2024, 2.3% (95% CI: 1.7–2.8) of Italian adults used EC, and 4.5% (95% CI: 3.7–5.2) HTP. Use of both products declined with age (p for trend <0.001). Dual use with CCs was prevalent among EC (87.8%; 95% CI: 80.1–95.4) and HTP (92.5%; 95% CI: 88.1–96.8) users. Compared with current CC smokers, odds of EC and HTP use were lower in never (EC: AOR=0.04; 95% CI: 0.02–0.09; HTP: AOR=0.01, 95% CI: 0.00–0.03) and former smokers (EC: AOR=0.14; 95% CI: 0.04–0.48; HTP: AOR=0.18; 95% CI: 0.08–0.41). Use of HTP increased by 40% over two years (p for trend = 0.085). Use of EC did not substantially change.

**CONCLUSIONS:**

The widespread dual use and the increasing prevalence of HTP use in Italy highlight growing public health concerns. Instead of serving as cessation aids for smokers, these devices are frequently used by youth and alongside CCs.

## INTRODUCTION

Tobacco use remains the world’s leading preventable cause of death, responsible for more than 8 million deaths each year^1,2^. In recent years, the industry has introduced novel nicotine delivery systems – electronic cigarettes (ECs), heated tobacco products (HTPs) and nicotine pouches – which are promoted as reduced-harm alternatives^3,4^. In Italy – one of the world’s first test markets for Philip Morris International’s IQOS (a HTP device that was first launched there and in Japan in 2014) – adult smoking remains high^5^, and uptake of EC and HTP has grown since they were introduced to the market^6^. Nicotine pouches are pre-filled sachets containing white nicotine powder, designed to be placed between the lip and gum for consumption^7^. Originally developed in Scandinavia, they are now being distributed globally by major tobacco manufacturers^7^. Monitoring the prevalence of these products and the determinants of their use in the Italian population is crucial in this context.

Despite the tobacco industry claims, a growing body of evidence from independent studies warns about the health effects of novel nicotine-containing products^2^, particularly when combined with conventional cigarette (CC) smoking (dual use)^8-10^. A recent systematic review concluded that EC use does not provide substantial harm reduction for all cigarette-caused diseases, while dual use poses greater health risks than CC smoking^8^. According to a recent qualitative risk assessment, ECs are likely to be carcinogenic to humans, with probable risks of lung and oral cancer^10^. A recent case-control study found that the addition of EC use to smoking accelerated the risk of developing lung cancer, with dual users having a fourfold increased risk of lung cancer compared with CC smokers^9^. Although clinical trials in controlled settings suggest that ECs can support quitting cigarette smoking, real-world evidence suggests that these devices do not improve smoking cessation rates. In fact, smokers who adopt ECs or HTPs are less likely to quit smoking than those who do not, and non-smokers who start using novel products are more likely to start CC smoking^11-15^. Of particular concern is the rapid uptake among adolescents and young adults, groups highly susceptible to nicotine addiction and potential ‘gateway’ progression, fueled by targeted marketing by the EC and the tobacco industry.

In Italy, novel tobacco and nicotine products are subject to substantially lower tax rates than CCs, resulting in a significant annual loss of tax revenue^16^. Furthermore, the indoor smoking bans established by Law 3/2003 (the Sirchia Law) do not apply to ECs and HTPs, permitting their use in bars, restaurants, on public transport and in other enclosed public spaces^17^. Advertising restrictions for ECs and HTPs are similarly weak. Unlike CCs, ECs and HTPs are not subject to a comprehensive advertising ban, and promotional campaigns often feature messaging and imagery that appeal to younger audiences^18^. This regulatory vacuum contributes to the normalization and spread of these products, particularly among young people. Findings from the Global Youth Tobacco Survey (GYTS) suggest that Italy is among the top countries for early experimentation with ECs, with more than half of adolescents aged 13–15 years having tried these products^19^. This concerning landscape highlights the importance of systematically monitoring both the prevalence and determinants of novel product use in the Italian population, as well as evolving trends in recent years. Therefore, this study aimed to provide updated national estimates of the prevalence and patterns of use of ECs and HTPs among Italian adults in 2024. We further investigated sociodemographic and behavioral determinants of their use and examined trends over the period 2022–2024.

## METHODS

The data used in this study come from a cross-sectional survey conducted in Italy between March and September 2024 by the Istituto di Ricerche Farmacologiche Mario Negri (Milan), with fieldwork carried out by BVA DOXA, the Italian branch of the Worldwide Independent Network/Gallup International Association (Milan). Regarding selection criteria, the sample was designed to be representative of the Italian population aged ≥15 years, including participants selected to reflect the national distribution by sex, age, geographical area, and socioeconomic characteristics. The Ethics Committee of Istituto Neurologico Carlo Besta–Milano acknowledged the collection of anonymous data in face-to-face population-based, observational, cross-sectional studies (File number 37, 2017).

The study used a multistage representative sampling approach to select participants, with recruitment process designed to preserve the representativeness of the final sample. Data were collected by trained interviewers in the context of a computer-assisted personal interview (CAPI) in participants’ house. Detailed methodology of the survey administration is described in previous articles^5^. The trend analysis included data from analogous DOXA surveys conducted in 2022 and 2023 in collaboration with the Italian National Institute of Health (Istituto Superiore di Sanità, ISS)^20,21^. These surveys used an analogous sampling and questionnaire methodology and were based on a representative sample of the Italian adult population.

The questionnaire collected information on the use of CCs, ECs and HTPs. Current CC smokers were defined as those who reported having smoked 100 or more cigarettes in their lifetime and who were currently smoking cigarettes or had quit within the past year. Never CC smokers were defined as individuals who reported having smoked fewer than 100 cigarettes in their lifetime. Former CC smokers were those who had smoked 100 or more cigarettes in their lifetime but had not smoked for at least one year. Individuals were categorized as EC users if they reported current use (daily or occasional) of any type of EC, including disposable, cartridge-based or rechargeable EC. Former EC users were those who reported having used e-cigarettes in the past, while never users were those who reported never having used or not knowing these products. For HTP use, individuals were categorized as users if they reported current use (daily or occasional) of any type of HTP, such as IQOS, glo or Ploom. Former HTP users were those who reported having used these products in the past, and never users were those who reported never having used or not knowing them. Data on the intensity of EC (puffs/day) and HTP (sticks/day) were also collected. Dual use was defined as the combined current use of CCs and ECs and/or HTPs. Poly use was defined as the combined current use of ECs, HTPs and CCs. The use of nicotine pouches was investigated among current CC smokers.

Sex was categorized as male or female. Age groups were defined as 15–29, 30–49, 50–64, and ≥65 years. Geographical area was classified into Northern, Central, and Southern Italy (including Islands). Participants’ level of education was classified as low for those who completed middle school or lower, intermediate for high school graduates, and high for university graduates or higher. The variables sex, age, education level, and smoking status were included as potential confounders.

### Statistical analysis

Statistical weights were generated and used in the data analysis phase to ensure representativeness of the Italian population aged ≥15 years. We used descriptive statistics, frequencies and percentages, and means and standard deviations, to analyze the variables. To assess the independent associations between selected sociodemographic characteristics and EC and HTP use, while controlling for potential confounders, we used multivariable unconditional multiple logistic regression models including terms for sex, age, education level, and smoking status. We estimated adjusted odds ratios (AOR) and corresponding 95% confidence intervals (CI) for EC use versus EC non-use, and HTP use versus HTP non-use. Statistical significance was defined as a p<0.05. All statistical analyses were performed using SAS (SAS version 9.4, SAS Institute) and R (R version 4.4.3).

## RESULTS

Detailed sociodemographic characteristics of the 3125 Italian adults (48.5% men and 51.5% women; mean age: 50.1 years) participating in the survey are provided in Supplementary file Table 1. No missing data were reported for the variables included in the analysis. In 2024, the prevalence of current cigarette smoking was 26.6% (95% CI: 25.0–28.1). Regarding novel product use, 2.3% (95% CI: 1.7–2.8) of Italian adults reported current EC use, and 4.5% (95% CI: 3.7–5.2) reported current HTP use (Table 1). Average daily intensity was 13.3 cigarettes/day (SD=6.0) for CC smokers, 40.2 puffs/day (SD=67.5) for EC users, and 9.3 sticks/day (SD=5.8) for HTP users.

**Table 1:** Tobacco and nicotine product use among Italian adults included in the study, overall and stratified by sex and age, Italy, 2024 (N=3125)

*Variables*	*Total* *% (95% CI)*	*Men* *% (95% CI)*	*Women* *% (95% CI)*	*15–29 years* *% (95% CI)*	*30–49 years* *% (95% CI)*	*50–64 years* *% (95% CI)*	*≥65 years* *% (95% CI)*
**Total,** n	3125	1515	1610	479	892	895	860
**Smoking status**							
Never	61.7 (60.0–63.4)	53.7 (51.2–56.2)	69.2 (67.0–71.5)	72.3 (68.3–76.3)	59.4 (56.2–62.6)	56.9 (53.6–60.1)	63.2 (60.0–66.4)
Former	11.7 (10.6–12.9)	15.1 (13.3–16.9)	8.5 (7.2–9.9)	1.6 (0.5–2.7)	9.2 (7.3–11.1)	12.6 (10.4–14.8)	19.1 (16.5–21.7)
Current	26.6 (25.0–28.1)	31.1 (28.8–33.5)	22.3 (20.3–24.3)	26.1 (22.2–30.0)	31.4 (28.4–34.5)	30.5 (27.5–33.5)	17.7 (15.2–20.3)
Intensity, mean cigarettes per day (SD)	13.3 (6.0)	14.0 (6.2)	12.4 (5.7)	10.8 (6.9)	13.4 (5.3)	13.4 (5.8)	14.9 (6.4)
**EC use**							
Never	95.5 (94.8–96.2)	94.3 (93.1–95.4)	96.6 (95.7–97.5)	93.9 (91.7–96.0)	93.3 (91.7–94.9)	95.9 (94.5–97.2)	98.3 (97.4–99.1)
Past	2.3 (1.7–2.8)	3.3 (2.4–4.2)	1.3 (0.7–1.8)	3.0 (1.4–4.5)	3.4 (2.3–4.6)	1.4 (0.6–2.1)	1.6 (0.7–2.4)
Current	2.3 (1.7–2.8)	2.4 (1.7–3.2)	2.1 (1.4–2.8)	3.2 (1.6–4.7)	3.3 (2.1–4.4)	2.8 (1.7–3.9)	0.2 (0.0–0.4)
Intensity, mean puffs per day (SD)	40.2 (67.5)	50.2 (77.8)	29.3 (54.3)	108.1 (126.1)	29.3 (42.8)	15.3 (13.3)	28.2 (33.7)
**HTP use**							
Never	93.9 (93.0–94.7)	92.9 (91.6–94.2)	94.8 (93.7–95.9)	89.0 (86.2–91.8)	90.5 (88.6–92.4)	94.7 (93.3–96.2)	99.2 (98.6–99.8)
Past	1.7 (1.2–2.1)	2.2 (1.4–2.9)	1.2 (0.7–1.8)	1.7 (0.5–2.9)	2.8 (1.7–3.9)	1.6 (0.8–2.5)	0.6 (0.1–1.0)
Current	4.5 (3.7–5.2)	5.0 (3.9–6.1)	4.0 (3.0–4.9)	9.3 (6.7–11.9)	6.7 (5.1–8.4)	3.6 (2.4–4.9)	0.3 (0.0–0.7)
Intensity, mean sticks per day (SD)	9.3 (5.8)	10.3 (6.2)	8.2 (5.2)	8.8 (5.9)	10.0 (6.0)	8.2 (5.3)	14.5 (4.3)

EC: electronic cigarette. HTP: heated tobacco product.

Among men, 2.4% (95% CI: 1.7–3.2) reported current EC use and 5.0% (95% CI: 3.9–6.1) reported current HTP use; among women, the corresponding figures were 2.1% (95% CI: 1.4–2.8) and 4.0% (95% CI: 3.0–4.9), respectively. By age group, current EC use was reported by 3.2% (95% CI: 1.6–4.7) of participants aged 15–29 years, 3.3% (95% CI: 2.1–4.4) of those aged 30–49 years, 2.8% (95% CI: 1.7–3.9) of those aged 50–64 years, and 0.2% (95% CI: 0.0–0.4) of those aged ≥65 years. For HTP use, the corresponding figures were 9.3% (95% CI: 6.7–11.9), 6.7% (95% CI: 5.1–8.4), 3.6% (95% CI: 2.4–4.9), and 0.3% (95% CI: 0.0–0.7), respectively.

**Figure 1:**
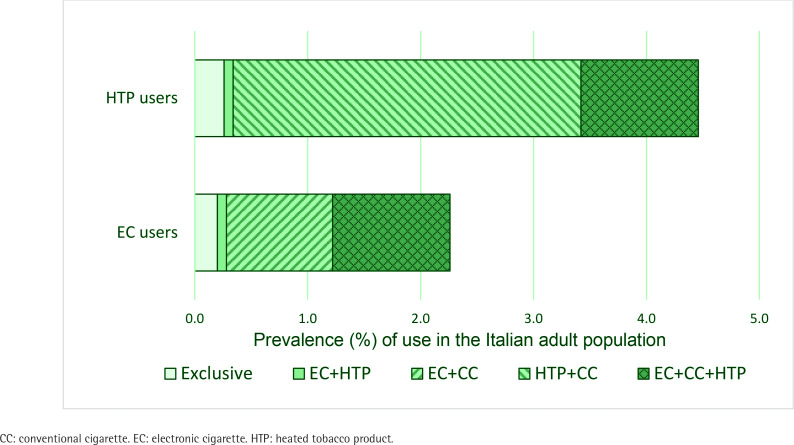
Prevalence of exclusive, dual, and poly-use among electronic cigarette and heated tobacco product users in the Italian adult population (aged ≥15 years), based on a representative sample of adults, Italy, 2024 (N=3125)

Among those reporting current EC use, 8.6% (95% CI: 2.1–15.2) used EC only, 3.6% (95% CI: 0.0–7.9) combined them exclusively with current HTP use, while 87.8% (95% CI: 80.1–95.4) were current dual users of ECs and CCs (46.0% were poly-users of ECs, HTPs and CCs) (Figure 1). Among HTP users, 5.7% (95% CI: 1.9–9.6) used HTPs exclusively, 1.8% (95% CI: 0.0–4.1) combined HTPs exclusively with ECs, and 92.5% (95% CI: 88.1–96.8) were dual users of HTPs and CCs (23.3% used all three products). Overall, 5.1% of Italian adults aged ≥15 years reported dual use of CCs with either ECs and/or HTPs. This prevalence was highest among those aged 15–29 years (9.3%) and 30–49 (7.7%), and lowest among those aged ≥65 years (0.3%). None of the CC smokers reported using nicotine pouches.

The multivariable analysis of factors related to current EC use and current HTP use is shown in Table 2. Increasing age was significantly related with a lower EC use (p for trend <0.001). Compared with the youngest age group (aged 15–29 years), the likelihood of EC use did not significantly differ for those aged 30–49 years (AOR=0.84; 95% CI: 0.43–1.61), and those aged 50–64 years (AOR=0.70; 95% CI: 0.36–1.37), but was significantly lower for those aged ≥65 years (AOR=0.06; 95% CI: 0.01–0.34). Geographical area was significantly associated with EC use, with lower odds observed in Central compared with Northern Italy (AOR=0.43; 95% CI: 0.19–0.98), while no significant difference was found between Southern/Islands and Northern Italy (AOR=0.95; 95% CI: 0.56–1.61). Both never and former smokers reported significantly lower EC use compared with current smokers (AOR=0.04; 95% CI: 0.02–0.09 for never smokers; AOR=0.14; 95% CI: 0.04–0.48 for former smokers). Finally, HTP users were more likely to use EC than HTP non-users (AOR=7.65; 95% CI: 4.36–13.42).

**Table 2:** Adjusted odds ratios (AORs) for current use of electronic cigarettes (ECs) and heated tobacco products (HTPs), according to selected characteristics, among Italian adults, Italy, 2024 (N=3125)

*Variables*	*EC users %*	*EC use vs EC non-use* *AOR (95% CI)*	*HTP users %*	*HTP use vs HTP non-use* *AOR (95% CI)*
**Total**	2.3	-	4.5	-
**Sex**				
Male ®	2.4	1.00	5.0	1.00
Female	2.1	1.28 (0.78–2.09)	4.0	1.34 (0.92–1.96)
**Age** (years)				
15–29 ®	3.2	1.00	9.3	1.00
30–49	3.3	0.84 (0.43–1.61)	6.7	**0.46 (0.29–0.74)**
50–64	2.8	0.70 (0.36–1.37)	3.6	**0.23 (0.14–0.39)**
≥65	0.2	**0.06 (0.01–0.34)**	0.3	**0.03 (0.01–0.11)**
p for trend		**<0.001**		**<0.001**
**Education level^a^**				
Low ®	1.3	1.00	1.7	1.00
Intermediate	2.4	0.92 (0.47–1.78)	5.6	1.71 (0.96–3.05)
High	3.6	1.34 (0.62–2.89)	6.3	**2.07 (1.06–4.05)**
p for trend		0.409		**0.039**
**Geographical area**				
Northern Italy ®	2.3	1.00	4.3	1.00
Central Italy	1.2	**0.43 (0.19–0.98)**	4.3	0.84 (0.51–1.41)
Southern Italy and Islands	2.8	0.95 (0.56–1.61)	4.8	0.80 (0.52–1.22)
**Smoking status**				
Never	0.3	**0.04 (0.02–0.09)**	0.2	**0.01 (0.00–0.03)**
Former	0.7	**0.14 (0.04–0.48)**	1.9	**0.18 (0.08–0.41)**
Current ®	7.5	1.00	15.5	1.00
**EC use**				
Non-user ®	-	-	3.4	1.00
User	-	-	49.8	**7.99 (4.53–14.11)**
**HTP use**				
Non-user ®	1.2	1.00	-	-
User	25.2	**7.65 (4.36–13.42)**	-	-

EC: electronic cigarette. HTP: heated tobacco product. AOR: adjusted odds ratio, estimated through unconditional multivariable logistic regression models adjusted for sex, age group, education level and smoking status. Statistically significant estimates at 0.05 level are reported in bold.

a Low for middle school or less, intermediate for high school graduates, high for university graduates or higher.

® Reference categories.

Compared with the youngest age group (aged 15–29 years), HTP use was less common among those aged 30–49 years (AOR=0.46; 95% CI: 0.29–0.74), among those aged 50–64 years (AOR=0.23; 95% CI: 0.14–0.39), and among those aged ≥65 years (AOR=0.03; 95% CI: 0.01–0.11), with a significant downward trend by age (p for trend <0.001). High education (university degree or higher) was related with higher HTP use compared to lower (middle school or lower) education level (AOR=2.07; 95% CI: 1.06–4.05; p for trend =0.039). Compared with current smokers, never smokers (AOR=0.01; 95% CI: 0.00–0.03) and former smokers (AOR=0.18; 95% CI: 0.08–0.41) reported significantly lower HTP use. Finally, EC users were more likely to use HTP compared with EC non-users (AOR=7.99; 95% CI: 4.53–14.11).

**Figure 2:**
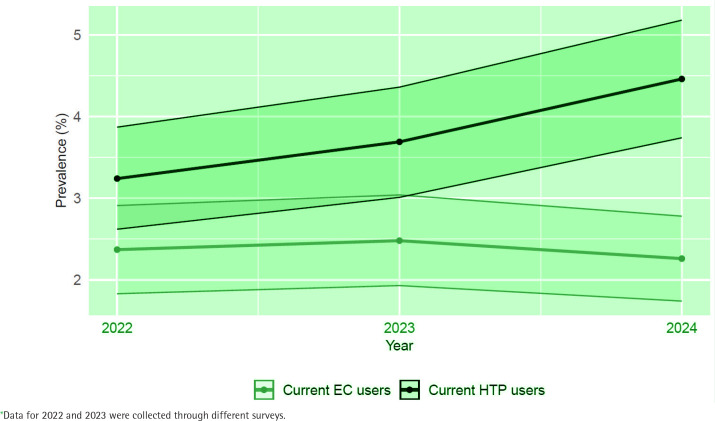
Prevalence trends, with 95% confidence interval, of electronic cigarette (EC) and heated tobacco product (HTP) current use in the Italian adult population (aged ≥15 years), based on a representative sample of adults, Italy, 2022–2024*

Figure 2 illustrates the trends in current use of ECs and HTPs over the last three years in the Italian adult population. Regarding EC use, the prevalence was 2.4% (95% CI: 1.8–2.9) in 2022, 2.5% (95% CI: 1.9–3.0) in 2023, and 2.3% (95% CI: 1.7–2.8) in 2024. For HTP use, the prevalence increased from 3.2% (95% CI: 2.6–3.9) in 2022 to 3.7% (95 % CI: 3.0–4.4) in 2023, reaching 4.5% (95% CI: 3.7–5.2) in 2024 (p for trend =0.085).

## DISCUSSION

This study provides an updated overview of novel tobacco and nicotine product use in Italy in 2024. The vast majority of EC and HTP users also smoke CCs, and the prevalence of dual and poly-use is high. These products are more prevalent among younger adults than among older adults. There has been a consistent, albeit not statistically significant, upward trend in the prevalence of HTP use, with an increase of 40% over the past two years. The use of ECs has been stable.

The large majority of EC and HTP users in the Italian adult population are current smokers, these products are predominantly used concurrently with CCs. In our sample, about nine out of ten EC and HTP users also smoked CCs, suggesting that these devices may not be functioning as intended cessation aids in real-world conditions, but rather as complementary nicotine sources. Emerging evidence suggests that dual users face greater health risks than those who only smoke CCs^8,9^. This widespread pattern of dual use raises concerns and highlights the need for policies to curb the spread of these products.

Our findings show that EC and HTP use is more common among younger age groups, with significantly lower use among older adults. These results are consistent with an increasing body of international literature suggesting that novel nicotine products are not primarily adopted by older smokers seeking cessation alternatives, but rather by younger individuals^12,22-24^. A recent systematic review of existing research on HTP use found that, in 15 studies, prevalence was consistently higher among individuals aged >40 years compared to older adults^12^. The widespread use of these products among adolescents and young adults is a pressing and concerning public health issue.

The tobacco industry claims that the overwhelming majority of HTP consumers are older adults, with over four out of five users reporting to be aged ≥30 years^25^. However, our data show that fewer than two in five EC users and approximately one in four HTP users in Italy are over the age of 50 years – an age group where hardcore smokers, i.e. those who could potentially benefit most from smoking cessation interventions, are concentrated. This discrepancy is further underscored by the notable absence of underaged kids in our cohort, given that independent evidence shows that significant numbers of adolescents and children use these products, also in Italy^26^. Therefore, if data on younger individuals had been included, the proportion of users aged >50 years would have been even smaller. These findings emphasize the discrepancy between industry assertions and independent epidemiological evidence.

The observed upward trajectory in HTP use, showing a marked relative increase in 2 years, signals a potentially widening public health challenge if left unaddressed. As these products are being adopted disproportionately by younger adults, continued market growth can be anticipated in the coming years, which would further entrench nicotine dependence in the Italian population. The growing popularity of HTPs could renormalize tobacco use and reverse decades of progress in tobacco control.

These products are not adequately regulated in Italy. Unregulated marketing, ranging from product advertising to social media promotions, explicitly targets adolescents and young adults, increasing curiosity and experimentation among youth and non-smokers^27,28^. There is a growing public demand for stronger regulation, with the vast majority of Italians in favor of extending existing smoke-free laws to novel nicotine products^29,30^. By contrast, countries such as Denmark and Finland already include ECs and HTPs in tobacco control measures, applying advertising bans, plain packaging, and flavor restrictions^31^. The European Commission has recommended extending smoke-free environments by banning CCs, ECs and HTPs also from outdoor areas, as part of the EU’s ‘Beating Cancer Plan’^32^. Therefore, Italian policymakers should priorities harmonizing regulations so that ECs and HTPs are treated equally to CCs.

None of the CC smokers reported using nicotine pouches. This result highlights that these products have not yet been adopted by adults in Italy. However, recent data from the European School Survey Project on Alcohol and Other Drugs (ESPAD) revealed that one in 25 students had used nicotine pouches in the past year (2024)^33^. Therefore, nicotine pouches seem to be just another nicotine product targeting youth and non-smokers instead of adult smokers in Italy. Further surveys should monitor nicotine pouch use in both the young and adult populations.

### Limitations

The present study has some limitations. Its cross-sectional design inherently precludes any inference of causality. The self-reported nature of the data introduces the potential for information bias, such as underreporting of smoking habits. However, the face-to-face interview approach with trained interviewers likely helped to minimize this bias. In addition, despite adjustment for key confounders, the possibility of residual confounding cannot be excluded. Another limitation of this study is that, for all tobacco and nicotine products, the exact time since cessation was not assessed, preventing a more accurate classification of former smokers and users. The modest number of EC and HTP users in our sample potentially constrained the power of some subgroup analyses. However, the sample was calibrated to mirror the Italian adult population, thereby enabling the findings to be generalized to the Italian population. Future research should consider representative samples of minors and adolescents to better characterize the prevalence and determinants of novel product use in younger age groups. The 2022 and 2023 surveys were conducted by a different research group; however, the questionnaire structure and key items were maintained to ensure comparability with the 2024 survey. The results on the prevalence of use may be affected by social desirability bias, whereby respondents tend to answer questions in a way that they perceive to be politically or socially correct. This can result in underreporting of self-reported behaviors relating to tobacco and nicotine use. In addition, since interviews with minors might have been conducted in the presence of an adult parent or relative, underreporting among younger participants cannot be ruled out. These biases may have led to an overall underestimation of the prevalence of use, suggesting that the actual consumption of these products – particularly among younger individuals – could be even higher.

## CONCLUSIONS

The growing prevalence of EC and HTP use – particularly among younger age groups – together with the alarmingly high rate of dual use with CCs observed in our data, strengthens concerns that these products may neither serve as effective cessation tools in Italy nor be primarily adopted by older adult smokers, contrary to tobacco industry claims^11-15^. Instead, they appear to be more commonly used by younger individuals, often alongside CCs. Further research, including longitudinal studies, is needed to clarify usage trajectories and potential health implications.

## Supplementary Material



## Data Availability

The data supporting this research can be found in the Supplementary file.
